# Pharmacological inhibition of protein tyrosine kinases axl and fyn reduces TNF-α-induced endothelial inflammatory activation *in vitro*

**DOI:** 10.3389/fphar.2022.992262

**Published:** 2022-12-01

**Authors:** Sophie F. Ellermann, Rianne M. Jongman, Matthijs Luxen, Timara Kuiper, Josee Plantinga, Jill Moser, Thomas W. L. Scheeren, Gregor Theilmeier, Grietje Molema, Matijs Van Meurs

**Affiliations:** ^1^ Medical Biology Section, Department of Pathology and Medical Biology, University Medical Center Groningen, University of Groningen, Groningen, Netherlands; ^2^ Department of Anaesthesiology, University Medical Center Groningen, University of Groningen, Groningen, Netherlands; ^3^ Perioperative Inflammation and Infection, Department of Human Medicine, Faculty of Medicine and Health Sciences, Carl von Ossietzky University Oldenburg, Oldenburg, Germany; ^4^ Department of Critical Care, University Medical Center Groningen, University of Groningen, Groningen, Netherlands

**Keywords:** endothelial inflammation, tumour necrosis factor alpha (TNF-α), postoperative organ damage, kinases, axl, fyn, BMS-777607, PP2

## Abstract

Major surgery induces systemic inflammation leading to pro-inflammatory activation of endothelial cells. Endothelial inflammation is one of the drivers of postoperative organ damage, including acute kidney injury Tumour Necrosis Factor alpha (TNF-α) is an important component of surgery-induced pro-inflammatory activation of endothelial cells. Kinases, the backbone of signalling cascades, can be targeted by pharmacological inhibition. This is a promising treatment option to interfere with excessive endothelial inflammation. In this study, we identified activated kinases as potential therapeutic targets. These targets were pharmacologically inhibited to reduce TNF-α-induced pro-inflammatory signalling in endothelial cells. Kinome profiling using PamChip arrays identified 64 protein tyrosine kinases and 88 serine-threonine kinases, the activity of which was determined at various timepoints (5–240 min) following stimulation with 10 ng/ml TNF-α in Human umbilical vein endothelial cells *in vitro*. The PTKs Axl and Fyn were selected based on high kinase activity profiles. Co-localisation experiments with the endothelial-specific protein CD31 showed Axl expression in endothelial cells of glomeruli and Fyn in arterioles and glomeruli of both control and TNF-α-exposed mice. Pharmacological inhibition with Axl inhibitor BMS-777607 and Fyn inhibitor PP2 significantly reduced TNF-α-induced pro-inflammatory activation of E-selectin, VCAM-1, ICAM-1, IL-6 and IL-8 at mRNA and VCAM-1, ICAM-1, and IL-6 at protein level in HUVEC *in vitro*. Upon pharmacological inhibition with each inhibitor, leukocyte adhesion to HUVEC was also significantly reduced, however to a minor extent. In conclusion, pre-treatment of endothelial cells with kinase inhibitors BMS-777607 and PP2 reduces TNF-α-induced endothelial inflammation *in vitro*.

## 1 Introduction

Surgical procedures induce a complex systemic inflammatory response. A key driver in the inflammatory response is the release of pro- and anti-inflammatory cytokines (Paruk and Chausse, 2019). An imbalance towards pro-inflammatory mediators leads to excessive inflammatory signalling, mounting in systemic inflammatory response syndrome (SIRS). SIRS can drive postoperative multi-organ dysfunction, including acute kidney injury (AKI). Patients suffer from AKI in up to 12% of noncardiac and up to 30% of cardiac surgery cases (Lei et al., 2019; Vives et al., 2019).

Endothelial cells line the vasculature and are at the first line of response to a surgery-induced excessive release of pro-inflammatory cytokines. Of these, Tumour Necrosis Factor-α (TNF-α) is acutely elevated postoperatively (Jongman et al., 2014). In endothelial cells, TNF-α initiates the expression of pro-inflammatory adhesion molecules, cytokines, and chemokines, allowing for leukocyte tethering, adhesion, and transmigration into underlying tissue, an important process in organ damage. TNF-α signalling is mediated by a complex network comprising of the nuclear factor ‘kappa-light-chain-enhancer’ of activated B-cells (NF-κB), mitogen-activated protein kinase (MAPK) and phosphoinositide 3-kinases (PI3K)-protein kinase B (AKT) pathways, which are also involved in many physiological processes (Choy et al., 2013; Sabio and Davis, 2014). Although anti-TNF-α drugs are available for inflammatory diseases such as rheumatoid arthritis, no treatment targeting TNF-α-induced signalling is available to mitigate endothelial pro-inflammatory activation in the postoperative context, blocking TNF-α-induced signalling completely, also in other cell types like macrophages in major surgery, is not seem desirable. Protein kinases are promising therapeutic targets to inhibit inflammatory activation, as they are essential components of many signalling pathways (Luxen et al., 2022). Kinases phosphorylate peptides on substrates in the presence of ATP in a covalent and reversible manner. This leads to temporary activation or inactivation of substrates, and subsequently results in biological effects such as increased adhesion molecule expression by endothelial cells (Liang et al., 2014).

To prevent excessive pro-inflammatory signalling in the postoperative context, pharmacological intervention is needed to rebalance pro- and anti-inflammatory signalling, without completely blocking TNF-α-induced signalling. By unravelling the nature and kinetics of kinase activity profiles induced by TNF-α in endothelial cells, we aimed to identify novel targets that upon inhibition can lead to reduced pro-inflammatory signalling and thereby prevent organ damage such as AKI in patients following surgery.

## 2 Materials and methods

### 2.1 Animals

Male C57Bl/6 mice were purchased from Harlan (Horst, Netherlands). Mice were randomly divided in experimental groups (n = 4). To mimic acute inflammation, after anaesthesia by inhalation of isoflurane/O_2_, 200 ng mouse TNF-α (Biosource Netherlands, Etten-Leur, Netherlands) in 20% bovine serum albumin/0.9% NaCl per mouse was intravenously injected in the orbital plexus. Control mice were injected with the vehicle only. All mice were sacrificed 2 h later, and kidneys were snap-frozen on liquid nitrogen and stored at -80 °C until analysis. All experimental procedures were approved and performed in compliance with the regulation of the local animal care and use committee of the University of Groningen.

### 2.2 Localisation of proteins in mouse kidney by immunohistochemical detection

Cryosections (4 µm) from snap-frozen mouse kidneys were fixed in acetone for 10 min at room temperature. Sections were encircled with a wax pen and rehydrated in phosphate-buffered saline (PBS). Endogenous peroxidases were blocked with 0.0375% hydrogen peroxide in PBS for 20 min.

E-selectin, VCAM-1, and ICAM-1 proteins: Primary antibodies (Table 1) were diluted in 5% (v/v) foetal calf serum (FCS) in PBS and sections were incubated 1 h at room temperature. After washing in PBS, sections were incubated for 45 min at room temperature with rabbit anti-rat antibodies (Vector Laboratories, Burlingame, CA, USA) supplemented with 1% normal mouse serum (Southern Biotech, Uden, Netherlands) in 5% FCS/PBS, followed by 30 min incubation with ready-to-use anti-rabbit-HRP polymer (Dako, Glostrup, Denmark).

**TABLE 1:** Primary antibodies and controls. IB - Immunoblot, IHC - Immunohistochemistry, IF - Immunofluorescence, FACS - Flow cytometry, * from Dr. Derek Brown, UCB Cell Tech, Belgium.

Antibody	Clone/Cat #	Supplier	Concentration used	Application
E-selectin	Mes-1	Kind gift *	10 μg/ml	IHC
VCAM-1	CBL1300	Merck Millipore	10 μg/ml	IHC
ICAM-1	YN1/1.7.4	ATCC	1:100 diluted supernatant	IHC
Phospho-NF-ĸB p65 (Ser536)	3,033	Cell signaling	0.06 μg/ml	IB
NF-ĸB p65	8,242	Cell signaling	0.5 μg/ml	IB, IF
Phospho-p44/42MAPK (Thr202/Tyr204)	9,101	Cell signaling	0.25 μg/ml	IB
p44/42 MAPK	9,102	Cell signaling	0.003 μg/ml	IB
Axl	4,566	Cell signaling	0.03 μg/ml	IB
Fyn	4,023	Cell signaling	0.4 μg/ml, 10 μg/ml	IB, IHC, IF
Lck	2,752	Cell signaling	0.2 μg/ml, 7.5 μg/ml	IB, IHC
GAPDH	sc-25778	Santa Cruz Biotechnology	0.04 μg/ml	IB
Axl	AF854	RnDsystems	2.5 μg/ml	IHC, IF
CD31	550,274	BDPharmingen	156 μg/ml	IF
APC CD106	305,810	BioLegend	3 µl in 100 µl staining volume	FACS
PE CD62E	322,606	BioLegend	3 µl in 100 µl staining volume	FACS
FITC CD54	322,720	BioLegend	3 µl in 100 µl staining volume	FACS
Rat IgG1	0,116-01	Southern Biotech	10 μg/ml	IHC
Rat IgG2a	0,117-01	Southern Biotech	10 μg/ml	IHC
Rat IgG2b	0,118-01	Southern Biotech	10 μg/ml	IHC
Rabbit IgG	0,111-01	Southern Biotech	10 μg/ml	IHC, IF
Goat IgG	02-6,202	Invitrogen	2.5 μg/ml	IHC, IF

AXL tyrosine kinase (Axl) protein: After washing, sections were blocked with 5% donkey serum (Jackson Immunoresearch, Ely, Cambridgeshire, UK), and subsequently incubated for 1 h at room temperature with primary antibody (Table 1) diluted in 5% FCS/PBS. Next, slides were incubated with ready-to-use VisUCyteHRP polymer Goat IgG antibody (R&DSystems, Abingdon, UK).

Fyn Src family tyrosine kinase (Fyn) and Leukocyte C-terminal Src kinase (Lck) protein: Primary antibodies (Table 1) were diluted in 5% FCS/PBS and sections were incubated 1 h at room temperature. After washing, slides were incubated with ready-to-use anti-rabbit-HRP polymer (Dako) for 30 min at room temperature.

Peroxidase activity in all sections was visualised with 3-Amino-9-ethylcarbazole (Sigma-Aldrich, Merck Life Science N.V. Amsterdam, Netherlands). All sections were counter stained with Mayer’s haematoxylin (Merck, Darmstadt, Germany) and mounted with Aquatex^®^ (Millipore, Amsterdam, Netherlands). After drying, slides were digitally scanned with Nanozoomer^®^ 2.0 HT (Hamamatsu Photonics, Almere, Netherlands).

### 2.3 Cell cultures of HUVEC and HL-60

Human umbilical vein endothelial cells (HUVEC) (CC2519, Lonza) were cultured in EBM-2 medium supplemented with EGM_2_MV SingleQuot Kit Supplements & Growth factors (Lonza) at 37°C and 5% CO_2_ by the Endothelial Cell Culture Facility of the UMCG. In all experiments, cells between passage 4-8 were used. HUVEC were seeded and grown overnight to a confluent monolayer.

For the leukocyte adhesion assays, the leukaemia cell line HL-60 (kindly provided by Dr. G. Fey, University of Erlangen, Germany) was cultured in RPMI 1640 medium (Lonza) supplemented with 10% (v/v) FCS.

### 2.4 Endothelial cell exposure to TNF-α for different durations

HUVEC were stimulated for 5, 10, 15, 20, 30, 60, 120 and 240 min with TNF-α (10 ng/ml) (Beromun^®^, Belpharma S.A. Luxembourg). HUVEC were lysed in Mammalian Protein Extraction Reagent lysis buffer (#78501) supplemented with 1% v/v Halt™ Protease inhibitor (#78415) and 1% v/v Halt™ Phosphatase Inhibitor (#78420, all reagents from Thermo Fisher Scientific, Bleiswijk, Netherlands) on ice. Total protein concentration was determined with BCA Protein assay kit (#23236, Thermo Fisher Scientific) according to manufacturer’s instructions.

### 2.5 Kinase profiling in endothelial cells

Protein samples were loaded on Protein Tyrosine Kinase (PTK, 5 µg/array) and Serine Threonine Kinase (STK, 1 µg/array) PamChip arrays (PamGene, ‘s-Hertogenbosch, the Netherlands) and processed according to manufacturer’s protocols (PTK: v.3.0; STK: v.5.1) using the PamStation12 (PamGene). Three independently generated biological replicates were used for both assays and values were normalised to unstimulated controls. The flow-through array tracks the phosphorylation of 196 and 144 peptides with the PTK and STK assays, respectively, based on which kinase activity is predicted. Post-array analyses were previously described by (Dayang et al., 2021). In brief, the BioNavigator software (v.6.3.67.0, PamGene) was used to acquire the read-outs mean specificity score (MSpS) and mean kinase statistics (MSK). For each time point, 25 kinases with the highest MSpS were evaluated and based on the highest activity (MSK), three kinases were selected for further analyses.

### 2.6 Immunoblot analysis of major signalling molecules and novel kinase targets

Cells were harvested 2 h after TNF-α stimulation by lysing in RIPA-buffer (50 mM Tris-HCl pH 8.0, 150 mM NaCl, 0.5% (w/v) sodium deoxycholate, 0.1% (w/v) sodium dodecyl sulfate, 1% (v/v) IGEPAL) containing cOmplete™ Mini Protease Inhibitor Cocktail (#04693124001) and PhosSTOP™ (#4906845001, all: Roche Diagnostics, Almere, Netherlands) for protein analysis. Total protein concentrations in lysates were determined with DC Protein assay according to manufacturer’s instructions (Bio-Rad Laboratories B.V. Veenendaal, Netherlands). Proteins were separated with SDS-PAGE on a 10% acrylamide gel (Bio-Rad), transferred to a nitrocellulose membrane (Bio-Rad), and blocked with 5% (w/v) skimmed milk (ELK, Campina^®^, Zaltbommel, Netherlands) in Tris (20 mM (w/v), 0.14 M NaCl (w/v), pH 7.5, Merck)-buffered saline-0.1% Tween (Sigma; TBS-T) for at least 1 h at room temperature. Membranes were probed overnight at 4 °C with primary antibodies in 5% BSA (w/v) in TBS-T (Table 1). Next, membranes were extensively washed with TBS-T and incubated with HRP-labelled secondary antibody in 5% ELK in TBS-T for 1 h at room temperature. Bands were visualised with Immobilon Forte Western HRP substrate (#WBLUF0500, Millipore) using GelDoc XR system (Bio-Rad). To assess phosphorylated and total protein levels of p65 and ERK1/2, membranes were first incubated as described above with the primary antibody against phospho-protein. Prior to repeating the blotting procedure with primary antibodies against total protein, membranes were incubated with PLUS all blot stripping buffer (Thermo Fisher Scientific) for 30 min at RT to remove the phospho-protein specific antibody. The signal was quantified and corrected for background using the image analysis software Quantity One (Bio-Rad). Normalisation of phosphorylated to total protein (for p65 and ERK1/2) was done. Fold-change values were calculated by normalisation to the control condition.

### 2.7 Co-localisation of proteins with endothelial cells in mouse kidney by immunofluorescence

Cryosections from snap-frozen mouse kidneys were fixed in acetone for 10 min at room temperature. Sections were encircled with a wax pen and rehydrated in PBS. Endogenous biotin was blocked with a Biotin Blocking system (Dako). Primary antibodies against Axl, Fyn, or CD31 (Table 1) were diluted in 5% (v/v) FCS in PBS and sections were incubated for 1 h at room temperature. After washing in PBS, sections were incubated with either AlexaFluor^®^555-conjugated rabbit anti-goat antibody (Thermo Fisher Scientific), AlexaFluor^®^488-conjugated goat anti-rat antibody (Thermo Fisher Scientific) to detect Axl, or biotin-conjugated goat anti-rabbit antibody (Southern Biotech, Uden, Netherlands) to detect Fyn, followed by AlexaFluor^®^555-conjugated streptavidin (Thermo Fisher Scientific) to stain CD31. All secondary antibodies were diluted in 5% FCS/PBS. After incubation, sections were washed in PBS and mounted in Aqua-Poly/Mount medium (Polysciences, Hirschberg an der Bergstrasse, Germany) containing 4′, 6-diamidino-2-phenylindole (Invitrogen, Leiden, Netherlands). Fluorescent images were taken with equal exposure times using a Leica DM4000 fluorescent microscope (Leica Microsystems, Germany) with Leica LAS V4.5 image software.

### 2.8 Pharmacological inhibition of target proteins in endothelial cells with selective small-molecule inhibitors

Selective small-molecule kinase inhibitors BMS-777607 (Selleck Chemicals, Breda, Netherlands), PP2 (Selleck Chemicals), or Dimethylsulfoxide (DMSO) control were added to HUVEC culture medium 30 min prior to TNF-α (10 ng/ml) stimulation for 2 h. Samples were harvested for the designated read-outs.

### 2.9 RNA isolation and gene expression analysis of endothelial adhesion molecules and cytokines by quantitative reverse-transcription (RTq)PCR

After experimental treatment, cells were washed with ice cold PBS (Lonza, Breda, Netherlands) and lysed in RLT-buffer containing 1% (v/v) β-Mercapto ethanol for mRNA expression analysis. Total RNA was isolated with the RNeasy Plus Mini kit (Qiagen) according to manufacturer’s instructions. Integrity of RNA was determined by gel electrophoresis, while yield (OD260) and purity (OD260/OD280) were measured with NanoDrop ND-1000 UV-Vis spectrophotometer (NanoDrop Technologies, Rockland, USA). Samples were reverse transcribed to complementary DNA using random hexamer primers (Promega, Leiden, Netherlands) and SuperScript III (Invitrogen, Breda, Netherlands). Next, 10 ng cDNA was used to perform quantitative PCR analysis using Assay-on-Demand primer/probe sets (TaqMan gene expression, Thermo Fisher Scientific) (Table 2) on a high throughput sequencer (ViiA7, Thermo Fisher Scientific). Obtained duplicate cycle threshold (CT) values were averaged for each sample. Gene expression was normalised to the expression of the housekeeping gene GAPDH, resulting in ΔCT value. The average mRNA levels relative to GAPDH were calculated by 2^−ΔCT^.

**TABLE 2:** Primer/probe sets used for mRNA expression analyses.

Gene	Assay id	Encoded protein
GAPDH	Hs99999905_m1	Glyceraldehyde-3-phosphate dehydrogenase (GAPDH)
PECAM1	Hs00169777_m1	platelet and endothelial cell adhesion molecule 1 (PECAM1), CD31
SELE	Hs00174057_m1	E-selectin, CD62E
VCAM1	Hs00365486_m1	Vascular cell adhesion molecule 1 (VCAM-1), CD106
ICAM1	Hs00164932_m1	Intracellular adhesion molecule 1 (ICAM-1), CD54
IL6	Hs00174131_m1	Interleukin 6 (IL-6)
CXCL8	Hs00174103_m1	C-X-C motif chemokine ligand 8 (CXCL8), IL-8

### 2.10 Cell surface expression of endothelial adhesion molecules by flow cytometry

After experimental treatment, medium was removed, cells were washed with PBS, trypsinised and collected in 5% (v/v) FCS in PBS. Cells were washed and centrifuged for 5 min at 1,500 rpm. Pellets were resuspended in 5% FCS/PBS and incubated on ice for 30 min with fluorescently-labelled primary antibodies (Table 1). After washing, cells were analysed using a Flow cytometer (NovoCyte Quanteon, Agilent Technologies Netherlands, Amstelveen, Netherlands)**.** Data was analysed with Kaluza (v2.1, Beckman Coulter).

### 2.11 Protein quantification of cytokines by enzyme-linked immunosorbent assay (ELISA)

After experimental treatment, the medium of the cells was collected and analysed for the presence of secreted IL-6 or IL-8 with ELISA MAX™ Standard Set Human IL-6 (cat no. 430501, BioLegend, London, United Kingdom) and IL-8 (#431501, BioLegend) according to the manufacturer’s instructions.

### 2.12 Leukocyte-endothelial cell adhesion assay by flow cytometry

After experimental treatment with or without pharmacological inhibitors 30 min prior to TNF-α for 2 h, medium was removed and HUVEC monolayers were incubated with 300,000 Hoechst-labelled (10 μg/ml, Life technologies) HL-60 cells in RPMI1640 supplemented with 5% (v/v) FCS for 1 h. Subsequently, the non-adherent HL-60 cells were removed by washing with RPMI1640, and the remaining adherent HL-60 cells and HUVEC were trypsinised and resuspended in 5% FCS in PBS. The percentage of adherent HL-60 cells over the number of total HL-60 and HUVEC cells in each sample was determined by flow cytometry (BD FACSVerse, BDBiosciences, Heidelberg, Germany). Experiments were carried out in three independent experiments and consisting of triplicate replicates per experimental condition. Due to technical issues in one experiment, one replicate of the ‘TNF-α’ condition and one of the ‘PP2 + TNF-α’ condition had to be excluded.

### 2.13 Statistical analysis

Data are presented as mean ± standard deviation from three independent experiments in triplicates. For each independent experiment, fold change was calculated for each sample and all experiments were plotted in one graph. Statistical analyses were performed using Mann-Whitney test in GraphPad Prism 9.0 (Prism Software Inca. La Jolla, CA, USA). Differences are regarded as statistically significant when *p* ≤ 0.05.

## 3 Results

### 3.1 Endothelial adhesion molecule expression is increased in vascular compartments of the kidney in TNF-α-exposed mice

**FIGURE 1:**
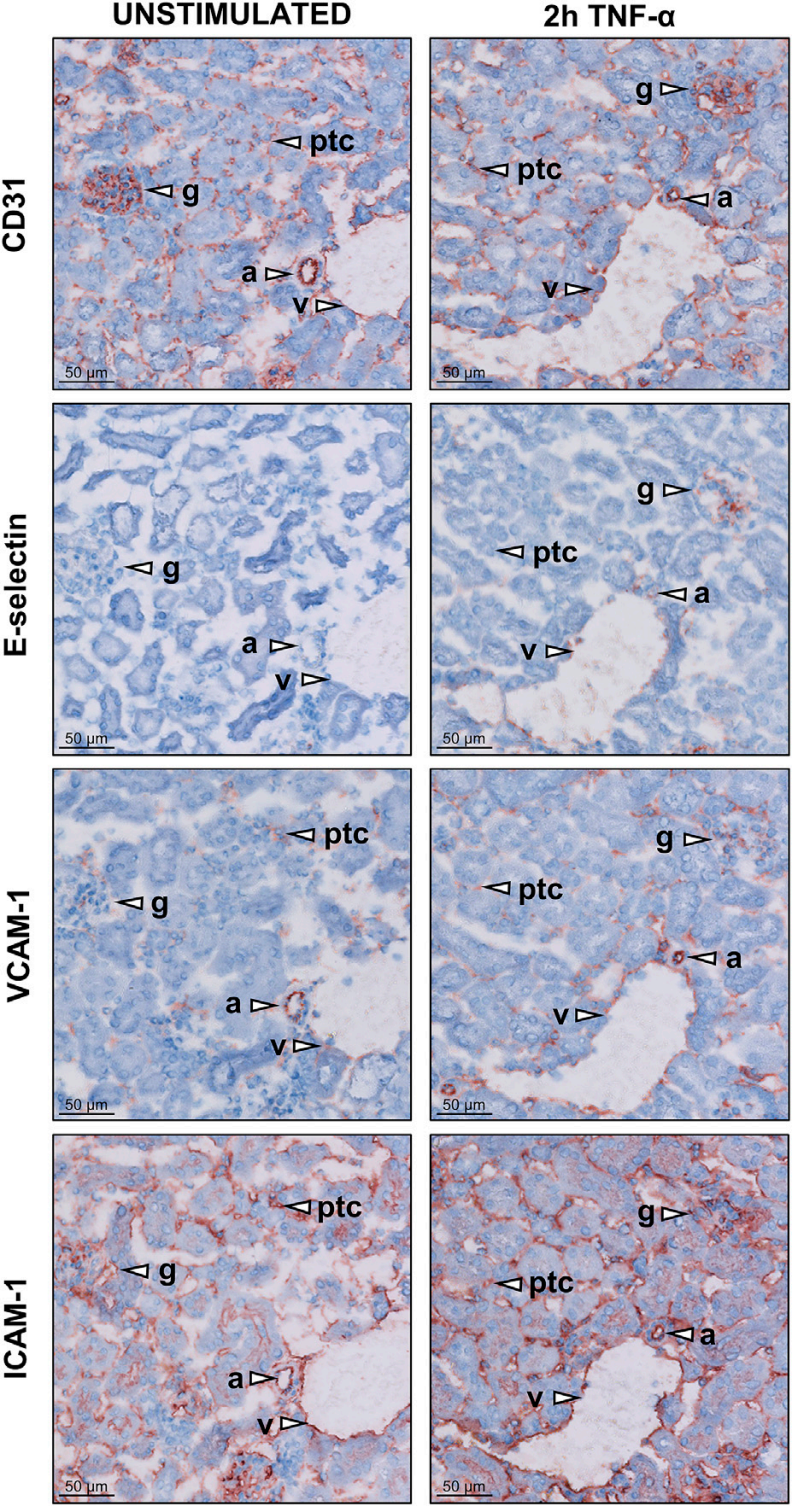
Expression of adhesion molecules in different microvascular compartments of the kidney in unstimulated control and TNF-α-exposed mice. Images of kidney cryosections of untreated mice and mice exposed to 200 ng TNF-α for 2 h showing the localisation of the endothelial marker molecule CD31 and the adhesion molecules E-selectin, VCAM-1 and ICAM-1. Representative images for each staining are shown. Arrowheads indicate different microvascular beds, arterioles (a), glomeruli (g), peritubular capillaries (ptc), and venules (v). Original magnification is ×200.

Surgical interventions trigger pro-inflammatory TNF-α release. To investigate the effect of TNF-α exposure on endothelial cell activation in mice, expression of adhesion molecules was determined in kidneys of these mice using immunohistochemistry. All renal microvascular compartments (arterioles, glomeruli, peritubular capillaries, and venules) stained positive for CD31, a marker of endothelial cells, in control mice and in mice sacrificed 2 h after TNF-α injection (Figure 1, S1). Staining for E-selectin was positive in arterioles, glomeruli, and venules in TNF-α-exposed mice, not in kidney of control animals, and not detectable in peritubular capillaries due to low tissue morphology quality. VCAM-1 staining was present in all microvascular compartments in control and TNF-α-exposed mice, with stronger staining of glomeruli in TNF-α-exposed mice compared to controls. ICAM-1 staining was present in all microvascular compartments, with no difference between both conditions. In conclusion, TNF-α exposure in mice resulted in pro-inflammatory activation of endothelial cells in different renal microvascular compartments.

### 3.2 Kinome analysis of TNF-α-stimulated endothelial cells show activity of known and novel kinases

**FIGURE 2:**
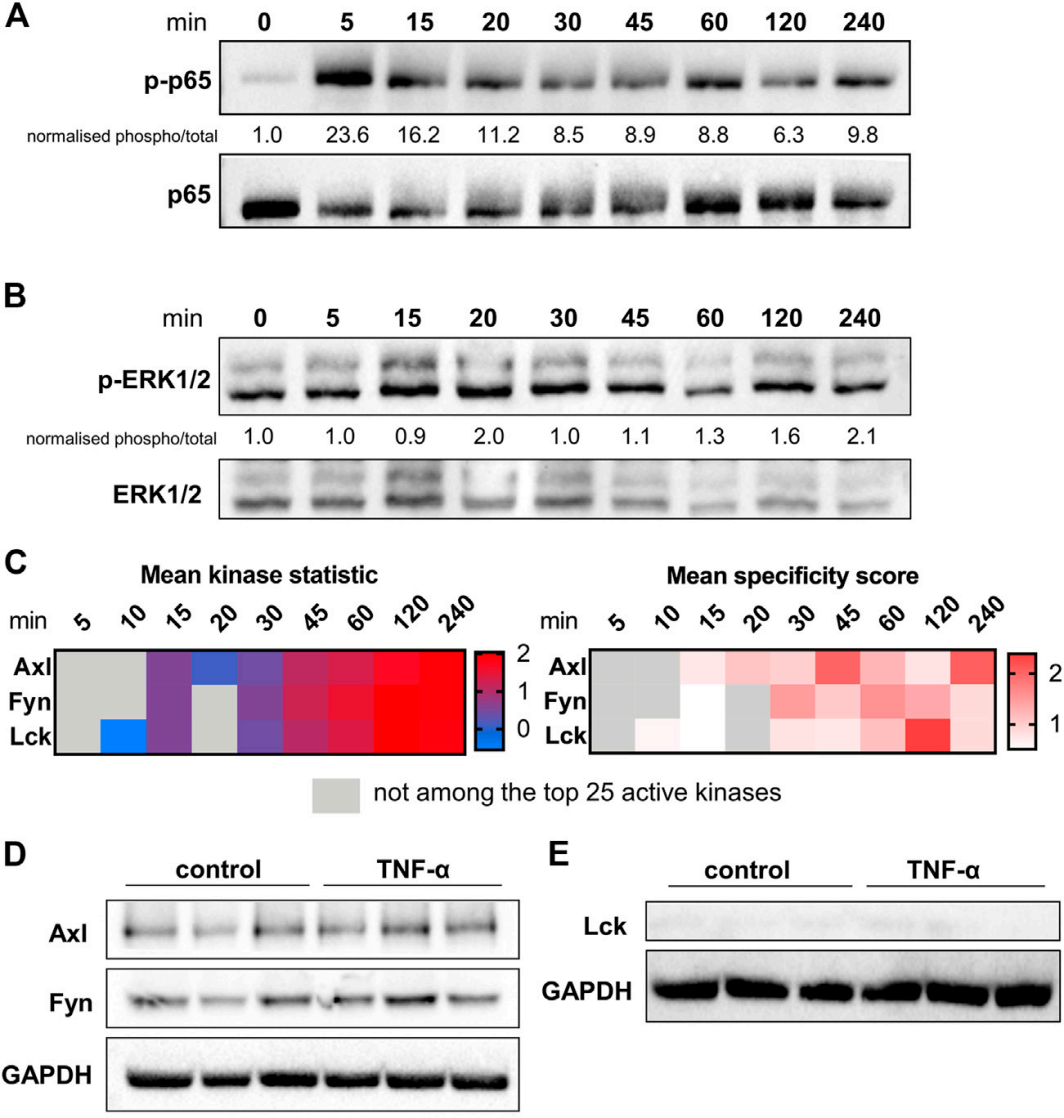
Kinase presence and activity in TNF-α-stimulated HUVEC. The influence of different durations of TNF-α (10 ng/ml) stimulation on the phosphorylation and total protein expression of **(A)** p65 and **(B)** ERK1/2 determined in HUVEC by immunoblotting. Quantification and normalisation of phosphorylated protein to total protein is shown as fold-change to unstimulated control. **(C)** Selection of three kinases as potential targets based on time-course data of the mean specificity score and mean kinase statistic (see Materials and Methods). The mean specificity score is based on peptide phosphorylation patterns relative to the unstimulated control condition and represents the likelihood of a kinase to be present (gradation of red). The mean kinase statistic indicates the predicted activity of the kinase relative to untreated control (gradation from blue, less active, to red, highly active). **(D)** Immunoblot of total HUVEC protein lysates showing presence of Axl and Fyn in control conditions or following 2 h TNF-α stimulation in HUVE. **(E)** Immunoblot of total HUVEC protein lysates showing absence of Lck in control conditions or following 2 h TNF-α stimulation in HUVEC. n = 3 independent experiments.

To identify targets for pharmacological inhibition of TNF-α-induced pro-inflammatory signalling, a kinome analysis was performed. First, the time course (5–240 min) of activation of known major signalling molecules p65, part of the NF-κB pathway, and ERK1/2, part of the MAPK pathway, was assessed at protein level in TNF-α-stimulated HUVEC (Figures 2A,B). Phosphorylation of p65 peaked at the early time point of 5 min after TNF-α exposure, whereas ERK1/2 phosphorylation was highest at the latest time point of 240 min, confirming the involvement of these signalling pathways in TNF-α signalling.

To identify specific kinases as potential novel targets in TNF-α-induced pro-inflammatory signalling in endothelial cells, the same HUVEC time course samples were analysed by PamChip arrays. Kinase presence, indicated by the MSpS, and kinase activity, shown by the MSK, were studied. Based on peptide phosphorylation profiles, kinases were identified at each time point after TNF-α stimulation relative to unstimulated control. Per time point, the 25 kinases with the highest MSpS were selected for further analyses. A distinct pattern of 64 differentially activated protein tyrosine kinases (PTKs, **Figure S2 A**) and 88 differentially active serine-threonine kinases (STKs, **Figure S2 B**) was observed between 5 and 240 min of TNF-α exposure. From this list of kinases, a selection of 34 PTKs and 42 STKs was chosen based on the MSpS being higher than one for at least one of the assessed time points. From this selection, kinases with highest activity (MSK) relative to control were identified. The potential target selection was narrowed down to kinases that have previously been detected in endothelial cells, and had been linked to inflammation, TNF-α-signalling or kidney damage. These kinases encompassed AXL tyrosine kinase (Axl), Fyn, and Lck, which were selected for further studies to test their role in TNF-α-induced pro-inflammatory signalling in endothelial cells (Figure 2). All three kinases exerted higher activity at 45–240 min compared to unstimulated control and were among the kinases with highest activity compared to control. Immunoblotting was used to validate the presence of Axl and Fyn in both unstimulated control and TNF-α-stimulated samples (Figure 2D) whereas Lck was not detected (Figure 2E) possibly due to a low level of expression in HUVEC.

### 3.3 Axl and fyn are expressed in renal microvascular compartments in mice

**FIGURE 3:**
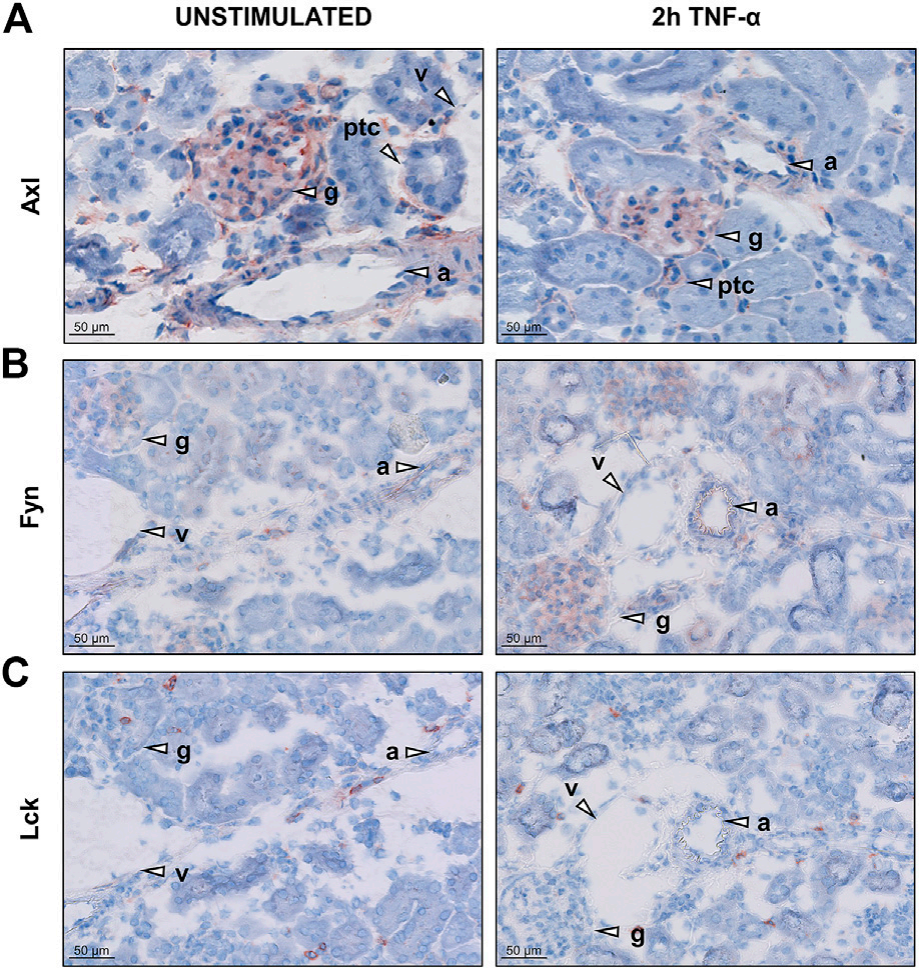
Renal microvascular expression of Axl, Fyn, and Lck in the kidney in unstimulated control and TNF-α-exposed mice. Images of kidney cryosections of mice exposed to 200 ng TNF-α for 2 h showing localisation of **(A)** Axl **(B)** Fyn and **(C)** Lck. Representative images are shown. Arrowheads indicate different microvascular beds, arterioles **(A)**, glomeruli (g), peritubular capillaries (ptc), and venules (v). Original magnification is ×200.

**FIGURE 4:**
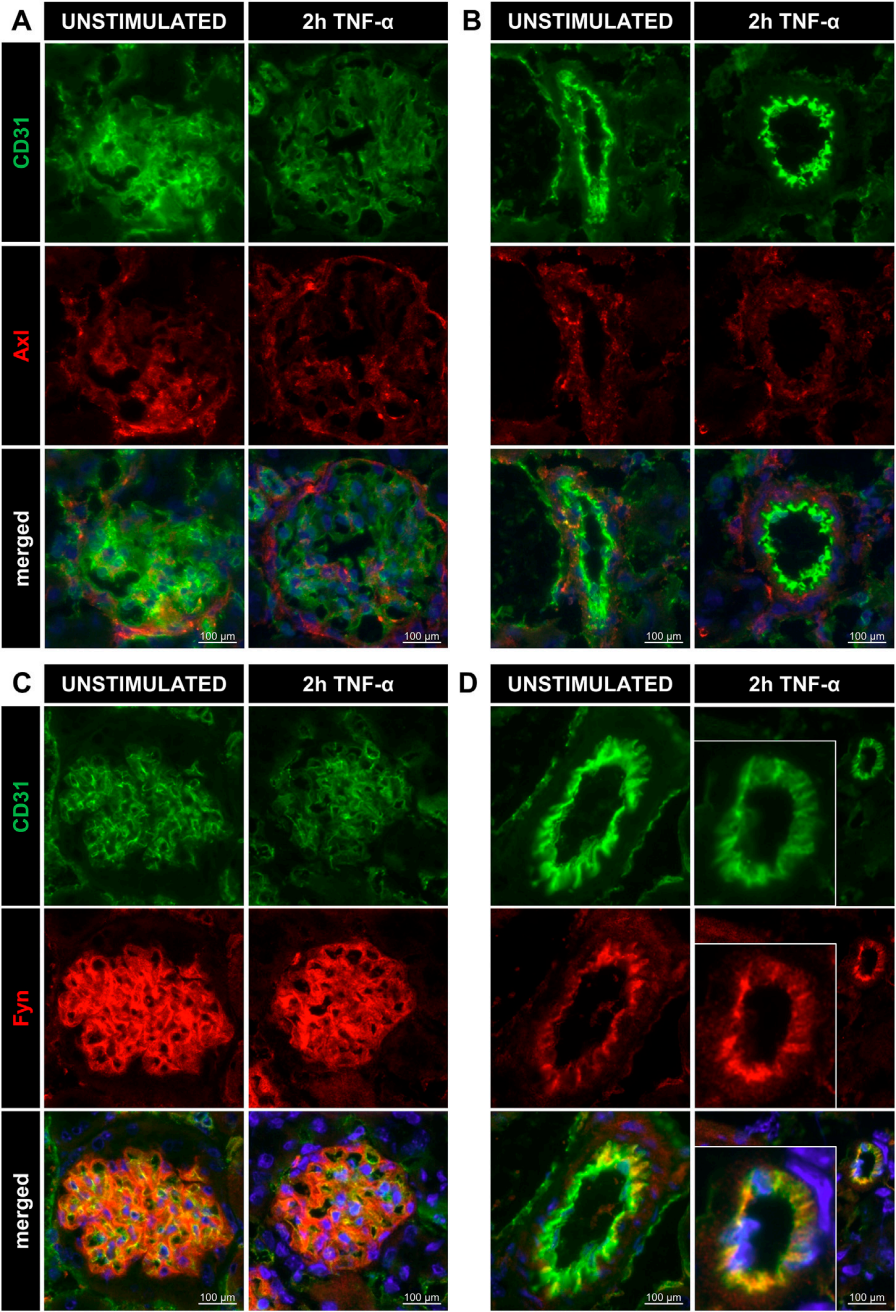
Renal microvascular localisation of Axl and Fyn in arterioles and glomeruli of TNF-α-exposed mice. Images of kidney cryosections of unstimulated control and 200 ng TNF-α-exposed mice showing staining for CD31 (endothelial cells, green), Axl (red), and DAPI (nuclear stain, blue) in glomeruli **(A)** and arterioles **(B).** Signal for CD31 (endothelial cells, green), Fyn (red), and DAPI (nuclear stain, blue) are shown in glomeruli **(C)** and arterioles **(D)**. Original magnification is ×400. For better visualisation, the arteriole was zoomed in on for the condition “2 h TNF-α”.

To further investigate the presence of the newly identified targets, we determined localisation of Axl, Fyn and Lck in renal vascular compartments. Kidney cryosections from control and TNF-α-exposed mice showed presence of Axl all renal microvascular compartments except for arterioles while Fyn was mainly expressed in arterioles and glomeruli (Figure 3, S3). Lck staining was negative for all microvascular compartments. Consequently, Lck was not included in further experiments of this study. To determine whether Axl and Fyn are expressed in endothelial cells, the endothelial-specific expression of Axl and Fyn was analysed by immunofluorescence co-staining with the endothelial marker CD31 (Figure 4). Co-localisation of Axl with CD31 was observed for some cells in glomeruli (Figure 4A), whereas the endothelial lining of arterioles did not express Axl (Figure 4B). Both arterioles and glomeruli showed strong co-localisation of Fyn and CD31 (Figures 4C,D), with arteriolar ECs increasing Fyn expression after TNF-α exposure. Thus, Axl and Fyn are expressed by endothelial cells in glomeruli of both control and TNF-α-exposed mice, while Fyn is also expressed in the endothelial lining of arterioles.

### 3.4 Pharmacological inhibition of axl and fyn attenuates TNF-α-induced expression of endothelial inflammatory molecules *in vitro*

**FIGURE 5:**
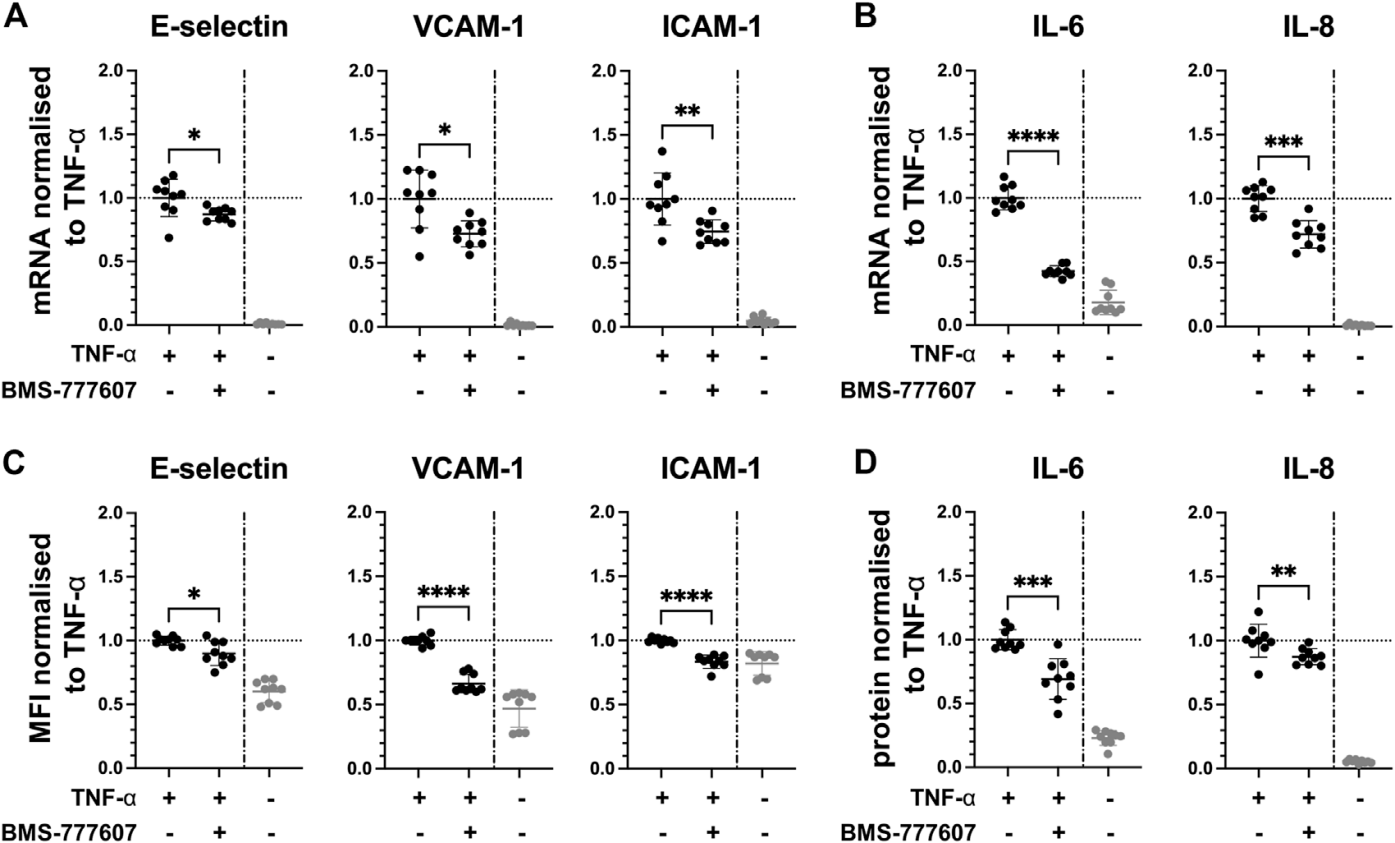
Pharmacologically inhibiting Axl pathway reduces pro-inflammatory responses in TNF-α-stimulated HUVEC *in vitro*. HUVEC were pre-treated with 2 µM BMS-777607 for 30 min prior to stimulation with TNF-α for 2 h **(A)** mRNA expression of adhesion molecules E-selectin, VCAM-1 and ICAM-1, and **(B)** pro-inflammatory cytokines IL-6 and IL-8 as measured with RT-qPCR. **(C)** Mean fluorescence intensity of cell surface expression of E-selectin, VCAM-1, and ICAM-1 measured by flow cytometry. **(D)** Protein expression of pro-inflammatory cytokines IL-6 and IL-8 released from TNF-α-activated HUVEC into the culture medium determined by ELISA. TNF-α vs. TNF-α + BMS-777607: ns = *p* > 0.05, * = *p* ≦ 0.05, ** = *p* ≦ 0.01, *** = *p* ≦ 0.001, **** = *p* ≦ 0.0001. N = three independent experiments.

**FIGURE 6:**
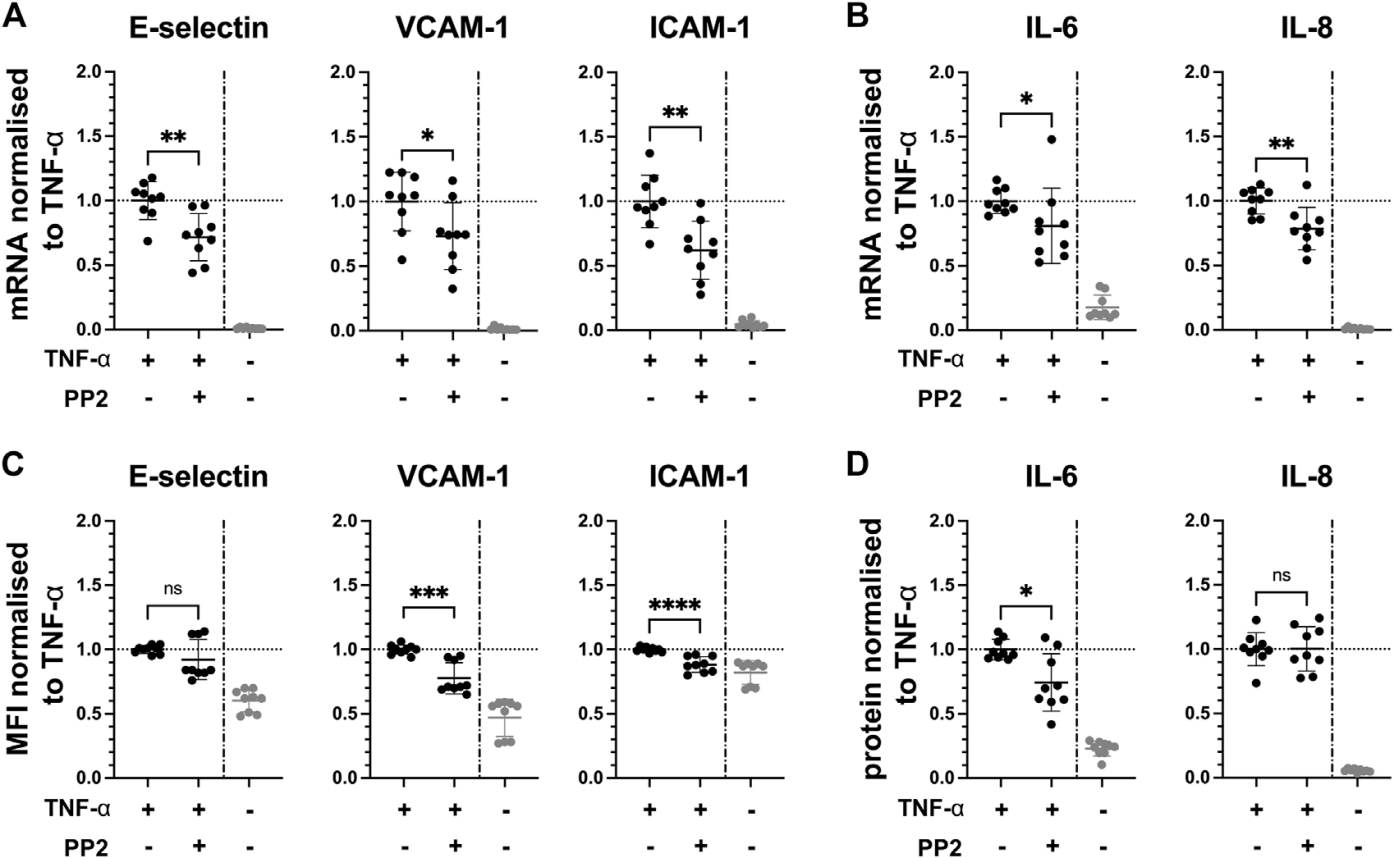
Pharmacologically inhibiting Fyn pathway reduces pro-inflammatory responses in TNF-α-stimulated HUVEC *in vitro*. HUVEC were pre-treated with 2 µM PP2 for 30 min prior to stimulation with TNF-α for 2 h. **(A)** mRNA expression of adhesion molecules E-selectin, VCAM-1 and ICAM-1, and **(B)** pro-inflammatory cytokines IL-6 and IL-8 as measured with RT-qPC. **(C)** Mean fluorescence intensity of cell surface expression of E-selectin, VCAM-1, and ICAM-1 measured by flow cytometry. **(D)** Protein expression of pro-inflammatory cytokines IL-6 and IL-8 released from TNF-α-stimulated HUVEC into the culture medium determined by ELISA. TNF-α vs. TNF-α + BMS-777607: ns = *p* > 0.05, * = *p* ≦ 0.05, ** = *p* ≦ 0.01, *** = *p* ≦ 0.001, **** = *p* ≦ 0.0001. N = three independent experiments.

Since the kinases Axl and Fyn are expressed in endothelial cells of some renal microvascular compartments, they can thus be considered potential targets for therapeutic intervention. We studied the effect of pharmacological inhibition of Axl and Fyn in endothelial cells *in vitro.* HUVEC were pre-treated with selective small-molecule kinase inhibitors for 30 min prior to TNF-α exposure for 2 h. In dose-response experiments, a dose 2 µM of BMS-777607 and PP2 was established for further use (**Figure S4 B**). The Axl inhibitor BMS-777607 attenuated TNF-α-induced mRNA and protein expression of E-selectin (mRNA = 11%; protein = 12%), VCAM-1 (mRNA = 26%; protein = 37%), ICAM-1 (mRNA = 26%; protein = 16%), IL-6 (mRNA = 58%; protein = 30%) and IL-8 (mRNA = 29%; protein = 13%; Figures 5A–D). At mRNA level, E-selectin (27%), VCAM-1 (26%), ICAM-1 (37%), IL-6 (23%) and IL-8 (23%) were also significantly reduced upon pre-treatment with the Fyn inhibitor PP2 (Figures 6A,B) compared to TNF-α. At protein level, VCAM-1 (29%), ICAM-1 (12%) and IL-6 (30%) were significantly reduced by PP2 (Figures 6C,D) compared to TNF-α while E-selectin and IL-8 were not. These results show that Axl and Fyn are partly responsible for TNF-α-induced endothelial pro-inflammatory activation.

### 3.5 BMS-777607 and PP2 reduce HL-60 adhesion to TNF-α-activated endothelial cells

**FIGURE 7:**
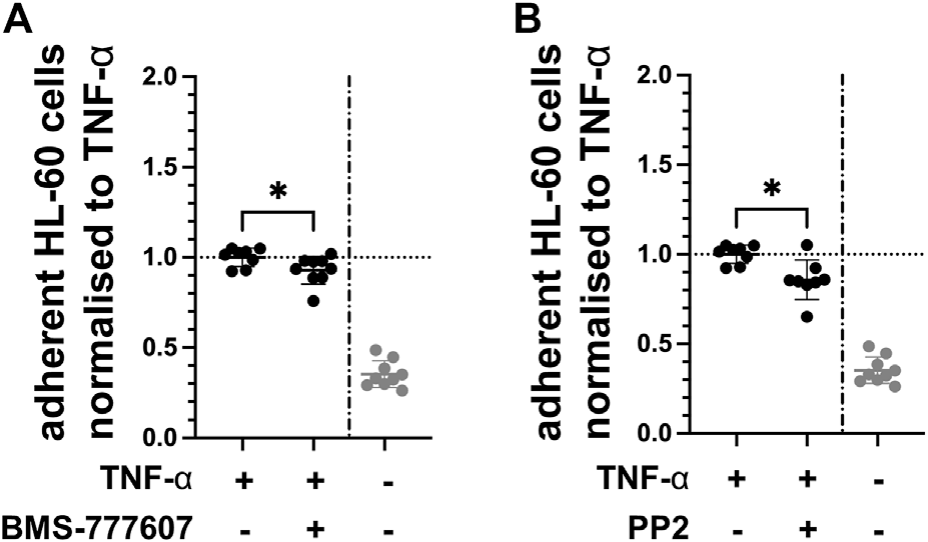
BMS-777607 and PP2 reduce HL-60 adhesion to TNF-α-activated endothelial cells. HUVEC were pre-treated with 2 µM **(A)** BMS-777607 or **(B)** PP2 for 30 min prior to stimulation with TNF-α for 2 h. Number of HL-60 leukocytes adherent to confluent monolayers of TNF-α-stimulated HUVEC were measured by flow cytometry. *p ≤ 0.05 compared to TNF-α stimulated. TNF-α vs. TNF-α + inhibitor: *p ≤ 0.05. N = three independent experiments.

Expression of endothelial adhesion molecules facilitates the adhesion of leukocytes, enabling the transmigration into tissue by these immune cells. Therefore, the effect of pharmacological inhibition of Axl and Fyn on the ability of leukocytes to adhere to TNF-α-activated endothelial cells was tested *in vitro*. The number of HL-60 leukocytes adhering to TNF-α-activated HUVEC was reduced by 7% with BMS-777607 (Figure 7A) and by 15% with PP2 (Figure 7B) compared to TNF-α. This indicates that the Axl inhibitor BMS-777607 and the Fyn inhibitor PP2 reduce the ability of leukocytes to adhere to endothelial cells.

## 4 Discussion

Acute inflammatory responses following surgery can induce organ damage such as AKI. Organ damage and AKI reduce the beneficial effects of surgery. Targeting protein kinases, which are common components of inflammatory responses downstream of pro-inflammatory mediators such as TNF-α, is a promising strategy to reduce disbalanced pro-inflammatory signalling after major surgery. The aim of this study was to discover kinases as novel targets for pharmacological inhibition of TNF-α-induced endothelial inflammation. Kinome analyses identified Axl, Fyn, and Lck as possible targets, and the presence of Axl and Fyn was confirmed in endothelial cells of microvascular compartments in kidneys of control and TNF-α-exposed mice. Pharmacological inhibition of Axl with BMS-777607 and Fyn with PP2 significantly prohibited TNF-α-induced endothelial pro-inflammatory molecule expression, suggesting the potential applicability of BMS-777607 and PP2 as pharmacological inhibitors in acute inflammation following surgery.

Axl is a receptor tyrosine kinase of the TYRO3 protein tyrosine kinase-Axl-Mer tyrosine kinase (TAM) family and a ubiquitously expressed proto-oncogene. In various cancer types, increased expression or activation of Axl contributes to tumour progression and treatment resistance by affecting various major signalling pathways (Wang et al., 2013; Park et al., 2015; Zhang et al., 2017; Grit et al., 2020; Hahn et al., 2021). In healthy tissue, TAM family members are generally known as negative regulators of inflammation, however, alterations have been reported in various pathological conditions, particularly in the context of innate inflammatory responses (Ni et al., 2019; Salmi et al., 2019; Van Beusecum et al., 2021). Previous studies suggest that inhibiting Axl has a therapeutic potential in murine nephritis and several different chronic human renal diseases (Zhen et al., 2016; Bian et al., 2021). In an *in vivo* model of chronic kidney disease, simultaneously increased Axl phosphorylation and nuclear translocation of phosphorylated p65 was reported in renal microvascular cryosections, suggesting a possible role of Axl in NF-κB signalling (Möller-Hackbarth et al., 2021). These data corroborate our data showing Axl expression in endothelial cells in glomeruli in control and TNF-α-exposed mice. Pharmacological inhibition of Axl was performed using the small-molecule kinase inhibitor BMS-777607, which has mainly been studied in oncological settings. In prior work of our group, we showed the involvement of Axl in LPS-activated endothelial cells and that BMS-777607 partially reduces LPS-induced endothelial activation (Dayang et al., 2021). In this current study, the administration of BMS-777607 30 min prior to TNF-α exposure resulted in significantly lower expression of adhesion molecules and cytokines at mRNA and protein level in HUVEC *in vitro*. The TNF-α-induced increase in adhesion molecule expression at protein level (Figure 5C), particularly ICAM-1, was almost completely abrogated by Axl inhibition. This finding corroborates the data from a study on the role of the Axl ligand Growth arrest-specific protein six in inflammation, which showed reduced expression of ICAM-1 upon knock-down of Axl in TNF-α-exposed HUVEC *in vitro* (Tjwa et al., 2008). Further, the number of leukocytes adhering to TNF-α-activated endothelial cells was reduced with kinase inhibitor administration, showing a functional response to pharmacological inhibition with BMS-777607 in TNF-α-stimulated HUVEC. However, the extent of reduction of leukocyte adhesion was minor compared to the substantial reduction in adhesion molecule expression. This may be due to the leukocyte adhesion assay having been performed under static conditions. The use of laminar shear stress would mimic the physiological *in vivo* condition better and may have resulted in lower baseline levels of adhered leukocytes as well as in the consecutive engagement of selectins and integrins resulting in a more realistic adhesion event. Also, the presence of DMSO in may have negatively impacted the rolling behaviour of HL-60 cells as it has been previously reported (Gee et al., 2012).

Fyn and Lck belong to the Src family kinases (SFKs) consisting of nine nonreceptor, membrane-associated tyrosine kinases (Ortiz et al., 2021). Research on SFKs in recent years elucidated differing roles and functional redundancies in various essential cell processes such as cell proliferation and cell migration, and pathologies such as cancer (Espada and Martín-Pérez, 2017). Eight SFKs were among the top 25 active kinases at multiple time points of TNF-α activation of HUVEC in our kinase screening (**Figure S2**), strongly suggesting their involvement in TNF-α signalling. Lck was not present in HUVEC or renal vascular compartments and thus, was not further investigated in our study. We showed presence of Fyn in endothelial cells*.* In pulmonary endothelial cells *in vitro*, Fyn has been shown to mediate nitric oxide production upon combined stimulation with TNF-α and LPS, indicating its role in inflammatory signalling (Chang et al., 2008). *Via* knockdown of Fyn, a study showed an association between Fyn and NF-κB signalling in endothelial cells *in vitro* (Freundt et al., 2022). In a review, the potential of Fyn as therapeutic target in AKI was highlighted, showing Fyn to be downstream of various receptors, i.e. TNFRs (Uddin et al., 2020). In two experimental studies, the same authors showed that inhibition of Fyn with the selective small-molecule kinase inhibitor PP2 prior to LPS or carbon monoxide stimulation effectively suppressed AKI. The authors suggested that this effect involves mitochondrial biogenesis (Pak et al., 2020) and reactive oxygen species-induced endoplasmatic reticulum stress in proximal tubular epithelial cells (Uddin et al., 2021). A role of Fyn in endothelial pro-inflammatory activation and leukocyte recruitment in AKI was not considered by these authors. In our study, we investigated this by pharmacologically inhibiting Fyn with inhibitor PP2 30 min prior to TNF-α exposure, showing significantly reduced expression of adhesion molecules (E-selectin, VCAM-1, ICAM-1) and cytokines (IL-6, IL-8) at mRNA level. At protein level, a significant reduction was also seen for VCAM-1, ICAM-1 and IL-6. A significant yet minor reduction in leukocyte adhesion to TNF-α-activated endothelial cells following PP2 pre-treatment highlights its functional relevance. However, as outlined above, this assay does not reflect the extent to which the adhesion molecules are reduced and further studies, *in vitro* with physiological laminar shear stress or *in vivo*, are required.

Using an unbiased screening method of kinases involved in TNF-α-induced pro-inflammatory signalling in endothelial cells enabled us to investigate differentially active kinases as novel therapeutic targets. However, our study has several limitations. First, we were not able to validate the effects of kinase inhibitors on the level of phosphorylated protein of Axl and Fyn, as respective antibodies did not work in our hands neither with immunoblotting nor with ELISA. Therefore, the molecular mechanisms underlying the inhibitory effects on endothelial inflammation were not established in detail. Second, other targets have been reported for the selective small-molecule kinase inhibitors we used. BMS-777607 also targets the TAM-family member TYRO3 protein tyrosine kinase, and other kinases, namely fms like tyrosine kinase 3, Macrophage-stimulating protein receptor, Met tyrosine-protein kinase and vascular endothelial growth factor receptor 2. Potential effects on endothelial inflammation through these other targets cannot be ruled out. PP2 is a selective inhibitor of SFKs, with highest affinity for Lck and Fyn. In HUVEC, we were not able to detect Lck at protein level. Therefore, we assume that the observed effects are exerted through Fyn, though contributions of other SFKs cannot be dismissed. Further, neutrophil and monocyte to endothelial cell adhesion are essential for initiating inflammatory processes in organs during and after major surgery *in vivo*. However, *in vitro* methods cannot mimic the *in vivo* complexity as for instance blood flow and, thus, shear stress, changes in local flow as observed during major surgery, and interactions with bloodborne cell types are lacking in these models. Lastly, exactly these *in vivo* studies are necessary to investigate the extent to which pharmacological inhibition with BMS-777607 and PP2 interferes with molecular processes vital for endothelial cells as well as other cell types under inflammatory stress.

In conclusion, we identified pharmacological targets in the TNF-α signalling pathway that may be further investigated to prevent pathological pro-inflammatory signalling in the postoperative context. Axl and Fyn showed increased activity upon TNF-α-exposure in HUVEC, reduced expression of pro-inflammatory endothelial molecules upon preventative pharmacological inhibition and are present in renal microvascular compartments. Further experiments more closely mimicking *in vivo* conditions are necessary to assess the therapeutic potential of the selective small-molecule kinase inhibitors against Axl and Fyn in postoperative acute kidney injury for patient benefit.

## Data Availability

The datasets generated for this study are stored in the repository figshare and can be accessed here: https://figshare.com/projects/Pharmacological_inhibition_of_protein_tyrosine_kinases_Axl_and_Fyn_reduces_TNF-_-induced_endothelial_inflammatory_activation_in_vitro/143142.
