# Supplementary figures and images for: Pharmacological inhibition of protein tyrosine kinases axl and fyn reduces TNF-α-induced endothelial inflammatory activation in vitro
Source: Front Pharmacol. 2022 Dec 1;13:992262. doi: 10.3389/fphar.2022.992262 (PMC9750991; doi:10.3389/fphar.2022.992262)

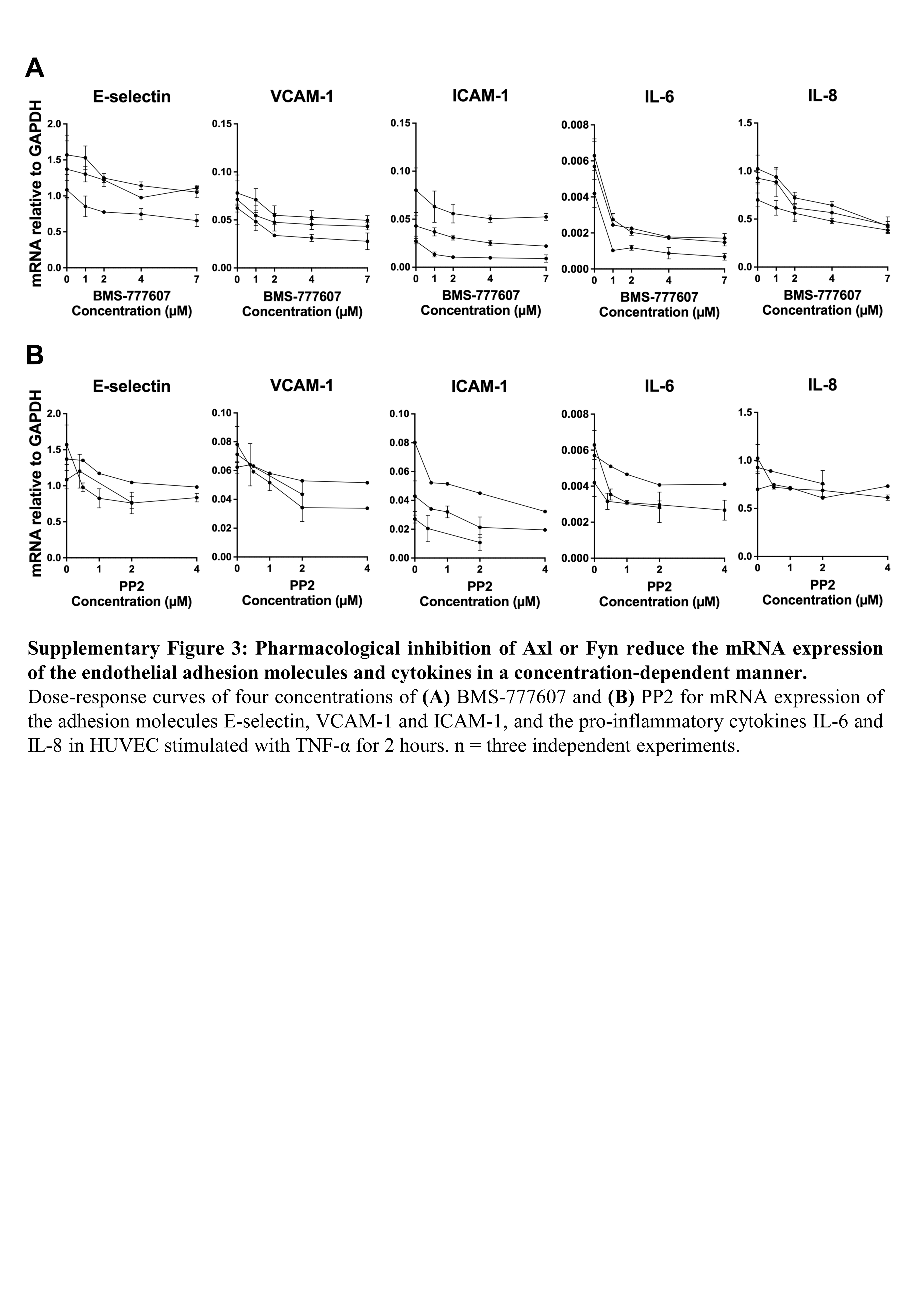

Supplement: Supplementary file 1 [file Image3.TIF]

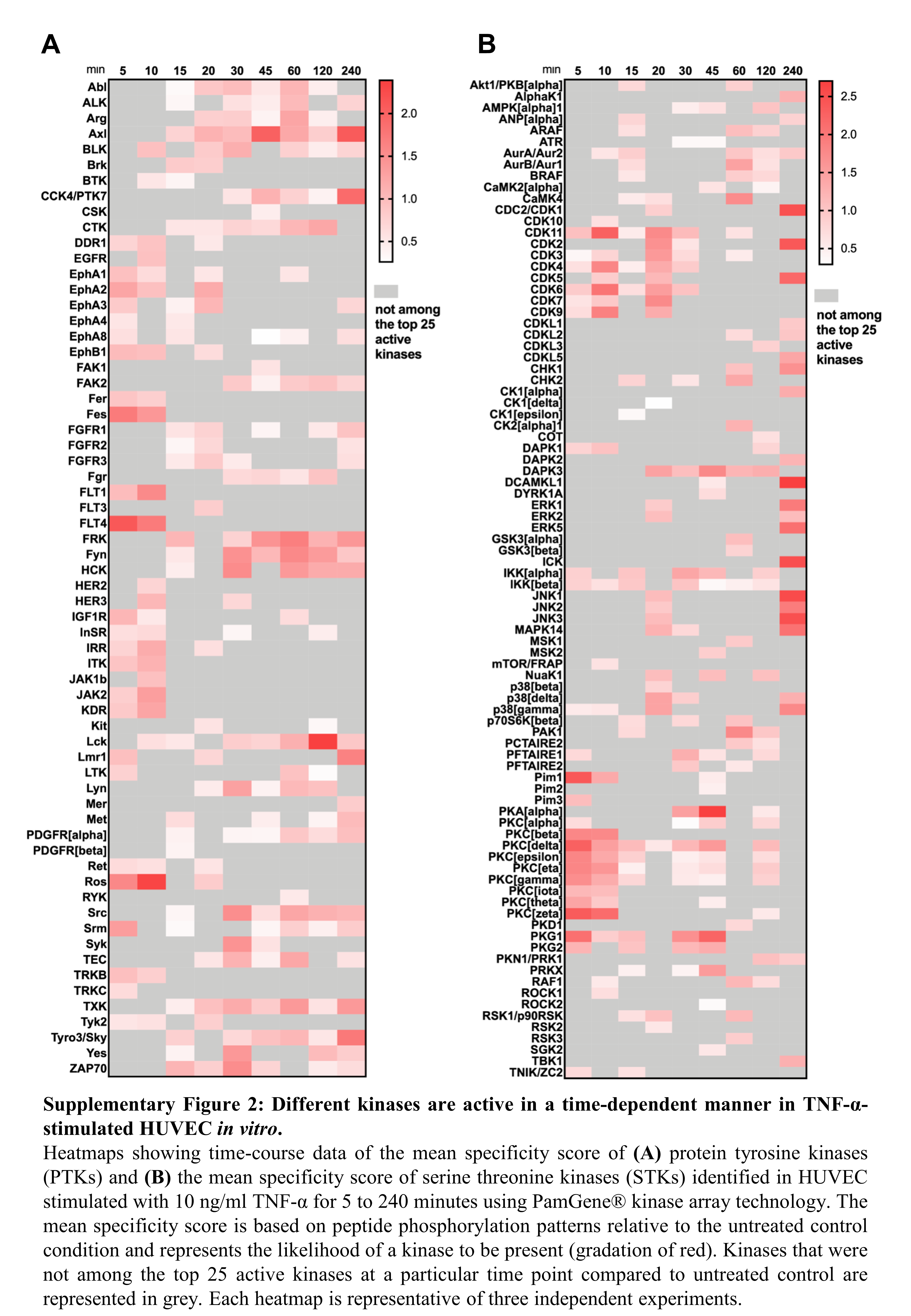

Supplement: Supplementary file 2 [file Image2.TIF]

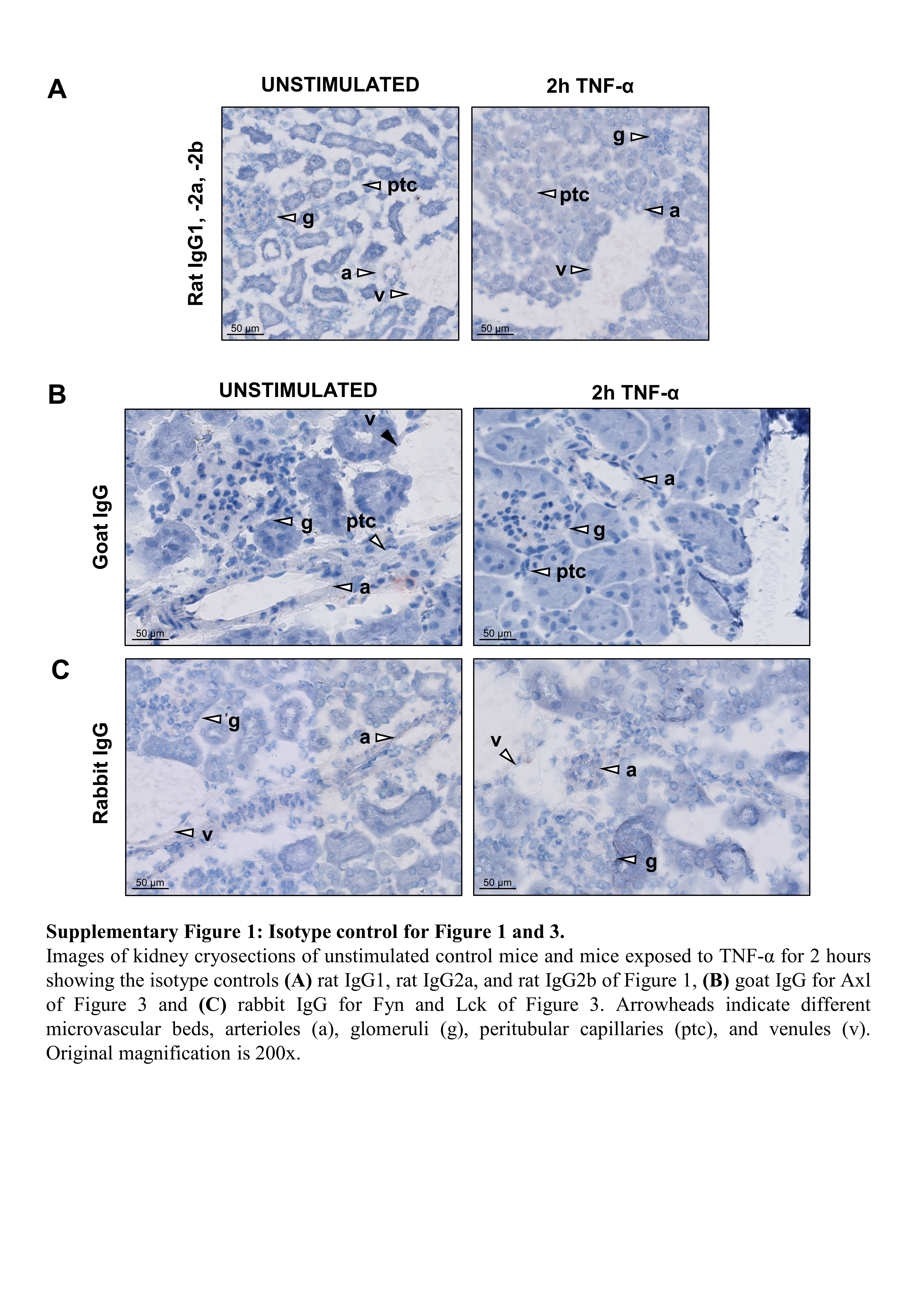

Supplement: Supplementary file 3 [file Image1.TIF]
